# Draft Genome Sequences of Seven Bacterial Strains Isolated from a Polymicrobial Culture of Coccolith-Bearing (C-Type) *Emiliania huxleyi* M217

**DOI:** 10.1128/genomeA.00673-16

**Published:** 2016-07-14

**Authors:** Albert Remus R. Rosana, Fabini D. Orata, Yue Xu, Danielle N. Simkus, Anna R. Bramucci, Yan Boucher, Rebecca J. Case

**Affiliations:** Department of Biological Sciences, University of Alberta, Edmonton, Alberta, Canada

## Abstract

Strains of *Rhodobacteraceae*, *Sphingomonadales*, *Alteromonadales*, and *Bacteroidetes* were isolated from a polymicrobial culture of the coccolith-forming (C-type) haptophyte *Emiliania huxleyi* strain M217*.* The genomes encode genes for the production of algal growth factors and the consumption of their hosts’ metabolic by-products, suggesting that the polymicrobial culture harbors many symbiotic interactions.

## GENOME ANNOUNCEMENT

The haptophyte *Emiliania huxleyi* is a coccolithophore, a phytoplankton that has the ability to make calcite disks (coccoliths)*.* Although it is possible to cultivate members of this species axenically, they are closely associated with bacteria in nature, and many commonly used cultures are polymicrobial. The *E. huxleyi* M217 and CCMP1516 strains both contain bacteria and represent isogenic lines isolated from the South Pacific in 1991. M217 can form coccoliths, but CCMP1516 has lost the ability to calcify (1). Bacteria associated with the former were isolated and their genomes sequenced to define its microbiota.

DNA was extracted from single-colony isolates using the DNeasy Blood and Tissue Kit (QIAGEN, Hilden, Germany) according to the manufacturer’s protocol. Sequencing libraries from the genomic DNA extracts were prepared using the Nextera XT DNA Library Preparation Kit (Illumina, San Diego, CA, USA). Whole-genome sequencing was performed using the NextSeq 500/550 High Output Kit version 2 (for 300 cycles) and NextSeq sequencing technology (Illumina), generating 150-bp paired-end reads. *De novo* assembly of the reads into contiguous sequences (contigs) was done using the CLC Genomics Workbench version 7.5.2 (CLC bio, Aarhus, Denmark). The draft genomes were then annotated using RAST version 2.0 (2) or PGAP (http://www.ncbi.nlm.nih.gov/genome/annotation_prok). All of the genomes sequenced exceeded 90× coverage, and the characteristics of the assemblies obtained are described in Table 1. Species identities were determined by an average nucleotide identity >95% using JSpecies version 1.2.1 (3), and genus identities were determined by an average amino acid identity (AAI) >60% using the AAI calculator (http://enve-omics.ce.gatech.edu/aai) (4) with previously sequenced genomes in the GenBank database. This analysis identified one *Bacteroidetes* isolate of the *Sphingobacteriales* order (*Balneola* sp. EhC07), one gammaproteobacterium of the *Alteromonadales* order (*Marinobacter* sp. EhC06), and three alphaproteobacteria from the *Roseobacter* clade (*Jannaschia* sp. EhC01, *Roseovarius indicus* EhC03, and *Sulfitobacter* sp. EhC04). *Rhodobacteraceae* bacterium EhC02 and *Sphingomonadales* bacterium EhC05 could not be attributed to a specific genus and were therefore named after the family and order to which they could be assigned, respectively.

**TABLE 1:** Genome features and GenBank accession numbers of the seven strains isolated from a polymicrobial culture of C-type *Emiliania huxleyi* M217

Isolate	Accession no.	Genome size (kb)	No. of contigs	*N*_50_ (kb)	G+C (mol%)
*Jannaschia* sp. EhC01	LXYJ00000000	4,580	96	98	62.7
*Rhodobacteraceae* bacterium EhC02	LXYH00000000	4,089	60	161	63.5
*Roseovarius indicus* EhC03	LXYQ00000000	5,512	168	81	64.8
*Sulfitobacter* sp. EhC04	LXYI00000000	4,853	89	162	61.3
*Sphingomonadales* bacterium EhC05	LXYP00000000	4,190	77	165	53.7
*Marinobacter* sp. EhC06	LXYO00000000	4,620	38	540	57.2
*Balneola* sp. EhC07	LXYG00000000	3,624	20	316	38.1

All the roseobacters encode genes for the ability to utilize the algal osmolyte dimethylsulfoniopropionate and to degrade lignin, a likely component of the *E. huxleyi* cell wall (5, 6). All bacteria have the ability to transport siderophores, but only one of them, *Marinobacter* sp. EhC06, has the ability to synthesize them. Like many other algal symbionts, all these bacteria encode a pathway to produce multiple vitamin Bs and the phytohormone auxin (7–9). This suggests a well-established symbiosis with their host, involving a production and consumption of a number of metabolites.

### Nucleotide sequence accession numbers.

The whole-genome shotgun projects have been deposited in DDBJ/ENA/GenBank under the accession numbers listed in Table 1.
